# Impacts of Organic Emerging Contaminants (Erythromycin, Ibuprofen, and Diclofenac) on the Performance of a Membrane Bioreactor Treating Urban Wastewater: A Heterotrophic Kinetic Investigation

**DOI:** 10.3390/membranes13080697

**Published:** 2023-07-27

**Authors:** Laura Antiñolo Bermúdez, Elena María Martínez Sánchez, Juan Carlos Leyva Díaz, María del Mar Muñio Martínez, Jose Manuel Poyatos Capilla, Jaime Martín Pascual

**Affiliations:** 1 Department of Civil Engineering, Institute of Water Research, University of Granada, 18071 Granada, Spain; lantinolo@ugr.es (L.A.B.); emms@correo.ugr.es (E.M.M.S.); jcleyva@ugr.es (J.C.L.D.); jpoyatos@ugr.es (J.M.P.C.); 2 Department of Chemical Engineering, University of Granada, 18071 Granada, Spain; mmunio@ugr.es

**Keywords:** erythromycin, ibuprofen, diclofenac, kinetic modelling, membrane bioreactor, respirometry, wastewater treatment

## Abstract

The occurrence of emerging organic contaminants, such as pharmaceuticals, is a growing global concern. In this research, for a membrane bioreactor (MBR) laboratory plant operating at a hydraulic retention time (HRT) of 24 h, fed with real urban wastewater, the heterotrophic biomass behaviour was analysed for two concentrations of erythromycin, ibuprofen, and diclofenac. The concentrations studied for the first phase were erythromycin 0.576 mg L^−1^, ibuprofen 0.056 mg L^−1^, and diclofenac 0.948 mg L^−1^. For Phase 2, the concentrations were increased to erythromycin 1.440 mg L^−1^, ibuprofen 0.140 mg L^−1^, and diclofenac 2.370 mg L^−1^. Heterotrophic biomass was affected and inhibited by the presence of pharmaceutical compounds in both phases. The system response to low concentrations of pharmaceutical compounds occurred in the initial phase of plant doping. Under these operating conditions, there was a gradual decrease in the concentration of mixed liquor suspended solids and the removal of chemical oxygen demand of the system, as it was not able to absorb the effect produced by the pharmaceutical compounds added in both phases.

## 1. Introduction

The occurrence of emerging organic contaminants (EOCs), such as chemicals in pharmaceuticals, plasticisers, and personal care products, in surface waters is an increasing global concern [1]. Pharmaceuticals represent one of the main groups of EOCs with adverse effects on living organisms, even at extremely low concentrations [2].

These pharmaceuticals are excreted as conjugates, but during treatment in wastewater treatment plants (WWTPs), the active moiety can be released by cleavage [3]. These compounds can be recognised as pseudo-persistent contaminants due to their repeated introduction into the environment through wastewater [4], and they can produce adverse effects in both animals and humans due to the presence of EOCs, among others, in water bodies [5]. Moreover, an additional concern for environmental and human health is related to the release of EOCs into soils, as well as their absorption by crops [6]. This makes it important to develop effective treatment strategies for the removal of EOCs.

According to Gros et al., the most frequently detected compounds in the basins of some rivers analysed in Spain are antibiotics (trimethoprim, sulfamethoxazole, erythromycin, and azithromycin), analgesics (ibuprofen, diclofenac, and naproxen), the antiepileptic carbamazepine, lipid regulators (bezafibrate and gemfibrozil), the blockers atenolol and sotalol, and the antihistamine ranitidine [4]. The total loads detected in river water concentration and in both the influent and the effluent are similar to those reported in other monitoring campaigns carried out in several countries such as Italy, Germany, Sweden, Canada, the United States, and Brazil [7,8].

The pharmaceuticals erythromycin, ibuprofen, and diclofenac were selected as focal compounds for this study. Notably, two of these compounds are on the EU watch list of emerging concern, namely erythromycin as an antibiotic compound and diclofenac as an anti-inflammatory compound. Furthermore, ibuprofen was included because of its widespread usage among the population.

Erythromycin (CAS 114-07-8) is a generic and inexpensive antibiotic of the macrolide family that inhibits bacterial protein synthesis. It is used as an alternative to penicillin for the treatment of infections caused by sensitive organisms [9]. Erythromycin molecules are resistant in the environment due to the structure of their aromatic ring, impeding their degradation or removal. At present, the presence of erythromycin in water and wastewater is above the standard level [10,11]. The effectiveness of the elimination of erythromycin in conventional wastewater treatment plants is 65.6% [12].

Ibuprofen (CAS 15687-27-1) is an anti-inflammatory pharmaceutical with an estimated annual global production of several kilotons; it has been identified in surface and wastewater (ng L^−1^), probably due to its stability regarding biodegradation and photolysis, associated with its hydrophobicity and low solubility in water (21 mg L^−1^) [13]. Ibuprofen has a 75% to 90% disposal rate in conventional wastewater treatment plants, one of the highest rates compared to those of other emerging contaminants [14].

Diclofenac (CAS 15307-79-6) is one of the most frequently detected pharmaceuticals in the aquatic environment. It is non-steroidal and anti-inflammatory, widely used as an antirheumatic, analgesic, and antiarthritic [15]. Because diclofenac biodegradation is limited, diclofenac removal rates in conventional WWTPs usually range from 21% to 40%, which results in the presence of diclofenac in aquatic organisms [16].

Generally, WWTPs for urban wastewater are based on conventional activated sludge. However, their ability to remove some of these compounds is discussed. Alternative technologies, such as the membrane bioreactor (MBR), are under extensive research, as they are presented as satisfactory treatment methods to improve the elimination of these compounds compared to conventional technologies in WWTPs [17]. The MBRs produce a higher effluent quality compared to conventional activated sludge systems, have a smaller ecological footprint, and can operate with a higher biomass concentration and sludge retention time (SRT) [18,19]. The MBR is an efficient technology for the treatment of urban wastewater because microfiltration or ultrafiltration membranes are used for biomass separation [20]. It has many advantages over the traditional activated sludge process for wastewater treatment, both in terms of process control and treatment effectiveness [21]. Other studies have focused on the degradation and removal of compounds of emerging concern (CEC) in different wastewater treatments, but there is little evidence of the effects of these compounds on heterotrophic biomass from MBR. Knowledge of this could improve the implementation of systems to obtain efficient degradation results [22]. Consequently, comprehending the distinct reaction patterns of heterotrophic biomass to pharmaceuticals holds potential for advancing the development of precise models and predictive tools [23,24,25]. Such advancements would facilitate the mitigation of pharmaceutical spill events and seasonal fluctuations in wastewater.

The aim of this study was to evaluate the effect of a mix of compounds of emerging concern (erythromycin, ibuprofen, and diclofenac) on an MBR with a hydraulic retention time (HRT) of 24 h and treating real urban wastewater, with the aim to determine the adaptive capacity of the biomass. For this purpose, the heterotrophic kinetics and organic matter removal efficiency were determined with two different concentrations of pharmaceuticals to evaluate the changes in the heterotrophic biomass.

## 2. Materials and Methods

### 2.1. The Laboratory Plant of the Membrane Bioreactor

**Figure 1:**
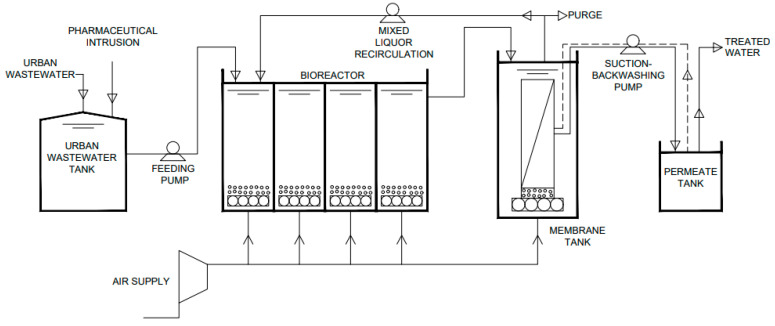
Flowchart of the implemented membrane bioreactor (MBR) system.

The laboratory plant was located in the Laboratory of Environmental Technologies of the University of Granada (Granada, Spain). To start the assays, the bioreactor was inoculated with mixed liquor from the Sur WWTP (Granada, Spain); the plant was fed with urban real wastewater from the primary settling tank of the Sur WWTP. The system is schematically represented in Figure 1.

The MBR consisted of an aerated rectangular bioreactor of four modules of 6 L each, which communicated with each other and worked as one through communicating vessels, with a total volume of 24 L and the following dimensions: 50 cm long, 12 cm wide, and 60 cm high. In the first model, the mixed liquor from the membrane tank was recirculated to keep the concentration of suspended solids inside the reactor constant. This module also contained a level probe to ensure that the volume in the bioreactor remained constant. The bioreactor outlet was connected to the cylindrical membrane tank with a diameter of 10 cm and a height of 65 cm, resulting in a total volume of 6.7 L and an effective volume of 4.32 L. It consisted of a vertically oriented module of hollow fibre microfiltration membranes. The total area of the membrane was 0.10 m^2^. The hollow fibres were made of polyvinylidene fluoride with a braided polyester inner reinforcement. They had an outer diameter of 2.45 mm, an inner diameter of 1.10 mm, and a pore size of 0.4 μm. The membrane tank was also connected to the aeration system, which constantly supplied a tangential air stream to prevent any organic or inorganic solids from depositing on its surface. Aeration was maintained at a constant rate to maintain a dissolved oxygen concentration of 2 mg/L.

### 2.2. Influent Characteristics

The laboratory plant was fed continuously with real urban wastewater from the Sur WWTP (Granada, Spain). After 13 days, the pharmaceuticals erythromycin, diclofenac, and ibuprofen were added to the feed tank.

For this research, different pharmaceutical compounds were added to the system in two different phases. For the first phase, a value 2.5 times lower than the water solubility value of erythromycin and diclofenac was established as a criterion for the amounts to be added. In the case of ibuprofen, due to its high solubility in water, the criterion established was a value 2.5 times lower than the maximum value detected in the wastewater in different areas of the world [26]. For the second phase, the criterion used to establish the amounts of the pharmaceuticals erythromycin and diclofenac was their water solubility value. In the case of ibuprofen, the maximum value detected in the wastewater was 55.97 µg/L [26]; this value was selected as the reference criterion for determining the minimum doping concentration of ibuprofen. Table 1 shows the concentrations used in this study.

### 2.3. Operation Conditions

The temperature at the laboratory plant was maintained at ±5 °C during the tests (15.5–20.5 °C) because the plant was located in the laboratory. The HRT was 24 h and remained constant throughout the phases. Under the plant operation conditions, cleaning and recovery of the membrane module with cyclic periods of filtration (9 min) and backwashing (1 min) were combined. A recirculation stream of activated sludge from the membrane chamber to the biological reactor was established to maintain the concentration of the heterotrophic biomass. This stream had a flow rate 50% higher than the influent stream. No increase in membrane module fouling and transmembrane pressure was observed. Consequently, there was no need for a cleaning and recovery cycle for the membrane modules. The membranes functioned efficiently and demonstrated a favourable level of permeability during operation. The working permeability values were maintained at 1.32–2.36 L/(m^2^ h bar). This working pressure is in the range of other studies working with membrane bioreactors in wastewater treatment with pharmaceuticals [27,28]. Table 2 shows the working permeability values.

The start-up of the laboratory plant, both in Phase 1 and Phase 2, was carried out by inoculating the plant with activated sludge from the Sur WWTP (Granada, Spain). For 13 days, the activated sludge was adapted to the new operation conditions. In both phases, the addition of the pharmaceutical mixture to the plant inlet water started on the 13th day of operation.

### 2.4. Analytical Determination

Samples from the influent, effluent, and bioreactor were periodically collected from the laboratory plant to characterise the wastewater. The tested properties were chemical oxygen demand (COD), biochemical oxygen demand on the fifth day (BOD_5_), pH, conductivity, and mixed liquor suspended solids (MLSS). The pH measurement was carried out with a Crison pH 25^®^ meter (Barcelona, Spain). Conductivity measurements were carried out with a Crison CM 35^®^ meter (Barcelona, Spain). Control analyses for BOD_5_, COD, and total suspended solids (TSS) were performed according to the standard methods [29].

### 2.5. Kinetic Analysis

For the study of the kinetic parameters of the heterotrophic biomass of the plant, respirometric tests were carried out in a BM-Advance respirometer of Surcis S.L. (Barcelona, Spain) The respirometer works at a stable temperature of 20.0 ± 1.0 °C, an aeration flow rate of 0.906 ± 0.001 L min^−1^, a pH of 7.25 ± 0.50, and a stirring speed of 2000 rpm. In addition, a recirculation stream was provided to ensure homogenisation of the sample in the respirometer.

One litre of heterotrophic biomass was taken from the laboratory plant for each of the assay pairs. This sample was homogenised by aeration for 24 h to ensure endogenous conditions. Once the biomass was homogenised, it was introduced into the respirometer, and two assays were carried out. The first one was the dynamic test with constant oxygen supply, where three additions of a 200 mg L^−1^ sodium acetate solution were made at increasing volumes (5, 10, and 15 mL). The second test was the static test in the absence of oxygen. These tests were carried out as described elsewhere [30].

For each litre of mixed liquor that contained the heterotrophic microorganisms, two respirometries were carried out consecutively. The first one was taken as a control to estimate the kinetic constants of the microorganisms. In the second respirometry, the mixture of pharmaceuticals was added to the sample in the concentrations that had been established for each phase. The aim of this was to determine the kinetic behaviour of the heterotrophic microorganisms in the mixed liquor and to check whether they have been able to adapt to the dodoping situation to which the laboratory plant was subjected by comparing the results obtained with those from the control respirometry. Table 3 lists the respirometric tests carried out for Phases 1 and 2.

To determine the kinetics of the analysed biomass, the kinetic constants were calculated as the yield coefficient of heterotrophic biomass (Y_H_), the maximum specific growth rate of heterotrophic biomass (µ_max_), the half-saturation coefficient of organic matter (K_M_), and the decay coefficient of heterotrophic biomass (b_H_). These constants were calculated following the kinetic parameter estimation of heterotrophic biomass described elsewhere [30]. The time, Rs values and OUR values were obtained for the different tests. Rs parameters provide insights into the aerobic capacity of microorganisms, allowing researchers to assess the respiratory performance and activity of biological systems, such as activated sludge used in this study. Monitoring and analysing the Rs in the respirometer can aid in evaluating treatment performance and understanding the kinetics of oxygen-consuming reactions in various biological systems. Unlike the Rs, the OUR parameter measures the rate of oxygen consumption by the biomass under static conditions. Subsequently, all kinetic constants were calculated, using the following equations:

- Oxygen consumption (OC) for each addition of sodium acetate:

$$\mathrm{OC}=\int_{\mathrm{ti}}^{\mathrm{tf}}{\mathrm{Rs}\;\mathrm{dt}\;,\;(\mathrm{mg}\;O}_{2}L^{-1})$$

- Yield coefficient for heterotrophic biomass (Y_H_):

$$Y_{H}=\frac{S-\mathrm{OC}}{S\;\cdot \;\mathrm{fcv}}\;(\frac{\mathrm{mgVSS}}{\mathrm{mgCOD}})$$

where: fcv: 1.48 mg COD/mg VSS (conversion factor)

S: substrate concentration (mgO_2_ L^−1^)

- Empirical specific growth rate for heterotrophic biomass (μ_emp_):

$$\mu_{\mathrm{emp}}=\frac{Y_{H}\;\cdot \;\mathrm{Rs}}{\left({1\;-\;Y}_{H}\;\cdot \;\mathrm{fcv}\right){\;\cdot \;X}_{H}}{\;(h}^{-1})$$

where: X_H_: concentration of heterotrophic biomass (mg VSS L^−1^)

- Linearisation of the Monod model:

$$\frac{1}{\mu_{\mathrm{emp}}}=\frac{1}{\mu_{m}}+\frac{K_{M}}{\mu_{m}}\cdot \frac{1}{S}\;(h)$$

where: K_M_: half-saturation coefficient of organic matter (mg O_2_/L)

- Decay coefficient of heterotrophic biomass (b_H_):

$$b_{H}=\frac{\mathrm{OURend}}{1.42\;\cdot \;X_{T}{\;\cdot \;[1-\;Y}_{H}\left(1\;-\;\mathrm{fp}\right)]}{\;(\mathrm{day}}^{-1})$$

where: X_T_: total biomass concentration biomass (mg VSS L^−1^)

(1 − fp): fraction of volatile biomass (mg VSS mg TSS^−1^)

- Substrate degradation rate of organic matter removal (r_su_):

$$r_{\mathrm{su}}=\frac{\mu_{m}{\;\cdot \;S\;\cdot \;X}_{H}}{Y_{H}{(K}_{M}+S)}\;\frac{{\mathrm{mgO}}_{2}}{L\;\cdot \;h}$$

## 3. Results and Discussion

Table 4 shows the results obtained for the laboratory plant.

For the influent, the total nitrogen values were 104.30 ± 29.84 mg N L^−1^, and the total phosphorus values were 11.42 ± 5.35 mg P L^−1^, given that the influent originated from a wastewater treatment plant with well-established stability previously analysed in other studies [30,31,32]. 

During both Phases 1 and 2, the MLSS values exhibited a declining trend. In Phase 1, the MLSS concentration showed a progressive increase from day 17 until day 30, followed by a distinct decrease of MLSS. Throughout Phase 1, the concentrations of pharmaceutical compounds remained lower compared to Phase 2, suggesting a lesser impact on the system. However, in Phase 2, the MLSS concentration decreased to 500 mg L^−1^ on day 35. Due to the operational characteristics of the system, there was no purge stream, and this caused the pharmaceuticals to bioaccumulate in the bioreactor as they were added to the particles of the mixed liquor, subjecting the biomass to considerable stress. Due to this, bulking of the heterotrophic biomass was observed, which led to a sharp decrease in the concentration of MLSS from day 30 onwards in Phase 1. The membrane exhibited optimal functionality throughout the two experimental phases, even during the bulking episodes. The loss of biomass from the system occurred as a result of tank overflow caused by bulking phenomena. This problem present in the laboratory plant is continued in the operation of wastewater treatment plants, not only in membrane bioreactor technology, but also in other configurations [33,34,35,36,37,38]. In Phase 2, the decrease in the MLSS concentration was due to pharmaceutical intrusion, where the system was not able to absorb the effect on the MLSS. Across the majority of experiments, it was observed that with increasing concentrations of pharmaceuticals, there was a corresponding rise in foam formation within the biological reactor. This phenomenon signifies the gradual impact of pharmaceuticals on the biomass. Nonetheless, a significant portion of the biomass became concentrated in the bulk. Consequently, the organic matter removal yields and the microbial kinetics exhibit this effect.

The COD removal capacity decreased in both phases of the study. The activity of the heterotrophic biomass decreased over time when the laboratory plant was fed with wastewater containing the pharmaceuticals. The performance decreased to values below 30% efficiency because only the COD fraction corresponding to the particulate COD was removed. This behaviour is likewise observed in the BOD_5_ removal rate. Upon the addition of pharmaceutical dosages to the plant, the microbial activity becomes constrained, leading to a reduction in BOD_5_ removal efficiency to 21% during Phase 1 and 14% during Phase 2. As Phase 2 involves higher pharmaceutical dosages, the overall performance experiences a more significant decline when compared to Phase 1.

**Figure 2:**
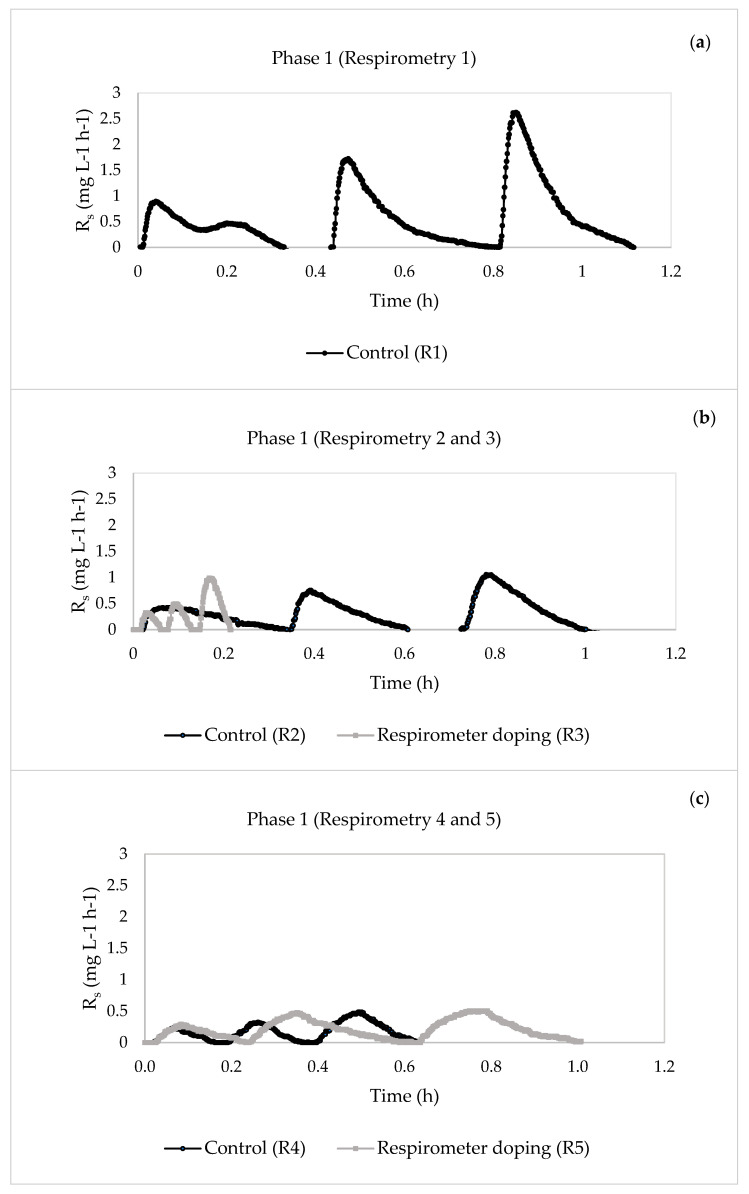

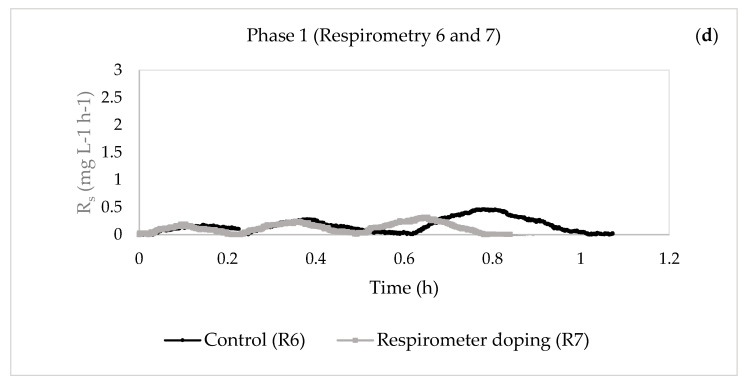
Evolution of the dynamic oxygen uptake rate (R_s_) in the respirometric experiments in Phase 1. (**a**) Phase 1: control (respirometric test 1). (**b**) Phase 1: control (respirometric test 2) and pharmaceutical compound addition in the respirometer (respirometric test 3). (**c**) Phase 1: control (respirometric test 4) and pharmaceutical compound addition in the respirometer (respirometric test 5). (**d**) Phase 1: control (respirometric test 6) and pharmaceutical compound addition in the respirometer (respirometric test 7).

**Figure 3:**
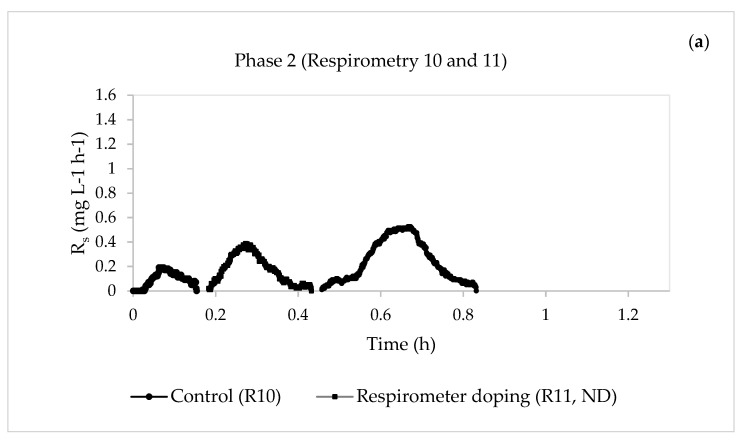

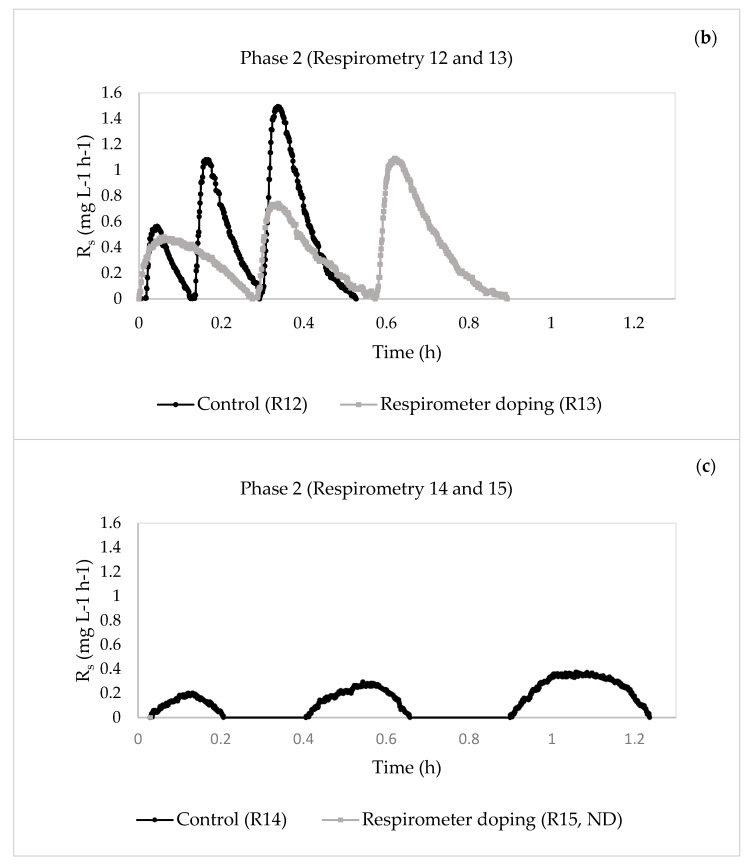
Evolution of the dynamic oxygen uptake rate (R_s_) in the respirometric experiments in Phase 2. (**a**) Phase 2: control (respirometric test 10) and pharmaceutical compound addition in the respirometer (respirometric test 11, not detected). (**b**) Phase 2: control (respirometric test 12) and pharmaceutical compound addition in the respirometer (respirometric test 13). (**c**) Phase 2: control (respirometric test 14) and pharmaceutical compound addition in the respirometer (respirometric test 15, not detected).

Figure 2 and Figure 3 show the results of the respirometric tests (exogenous respiration) performed for the two phases.

As seen in Figure 2, the amounts of the pharmaceuticals decreased during the respirometric tests, except during respirometric test 5 (Figure 2c), where it increased from 0.63 to 1.01 h. In general, the presence of this mixture of pharmaceuticals reduced the three maximum values of R_s_ (Figure 2b,d and Figure 3b), regardless of the pharmaceutical concentration and the method of compound addition (influent of the laboratory plant or in the respirometer), except for respirometric test 5 (Figure 2c).

**Figure 4:**
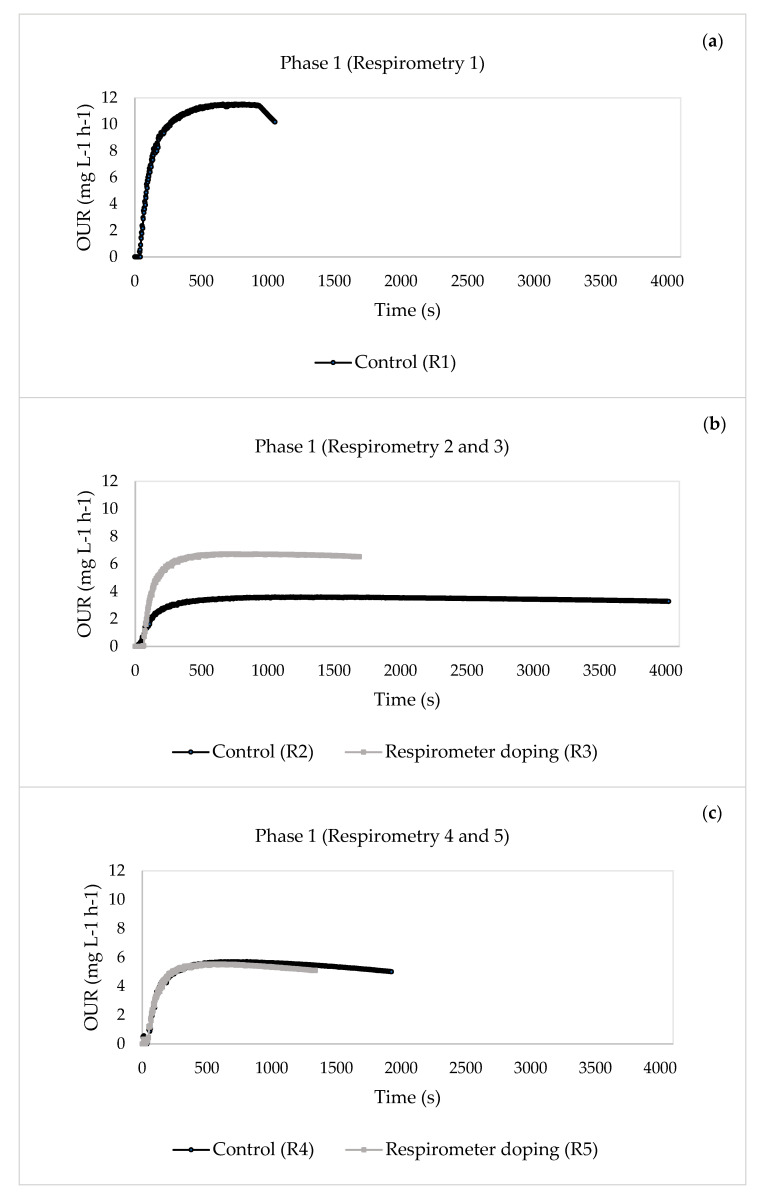

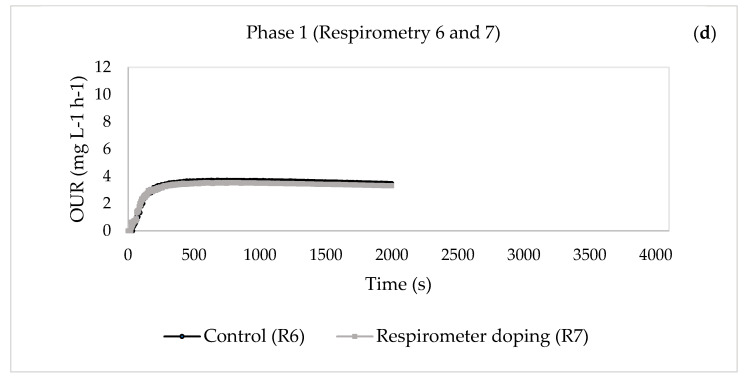
Evolution of the static oxygen uptake rate (OUR) in the respirometric experiments in Phase 1. (**a**) Phase 1: control (respirometric test 1). (**b**) Phase 1: control (respirometric test 2) and pharmaceutical compound addition in the respirometer (respirometric test 3). (**c**) Phase 1: control (respirometric test 4) and pharmaceutical compound addition in the respirometer (respirometric test 5). (**d**) Phase 1: control (respirometric test 6) and pharmaceutical compound addition in the respirometer (respirometric test 7).

**Figure 5:**
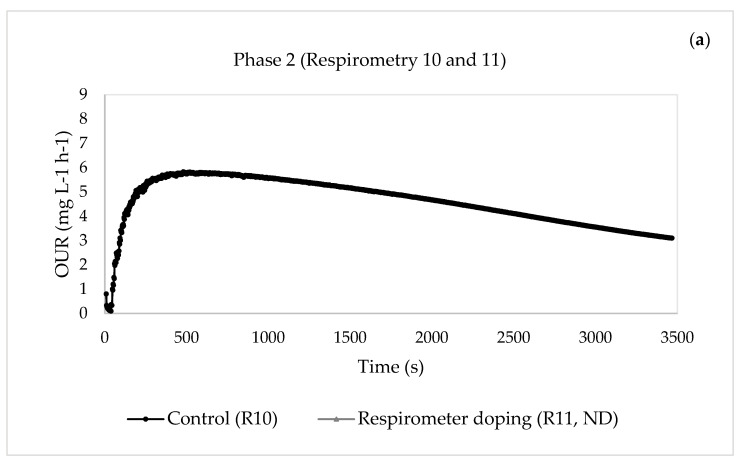

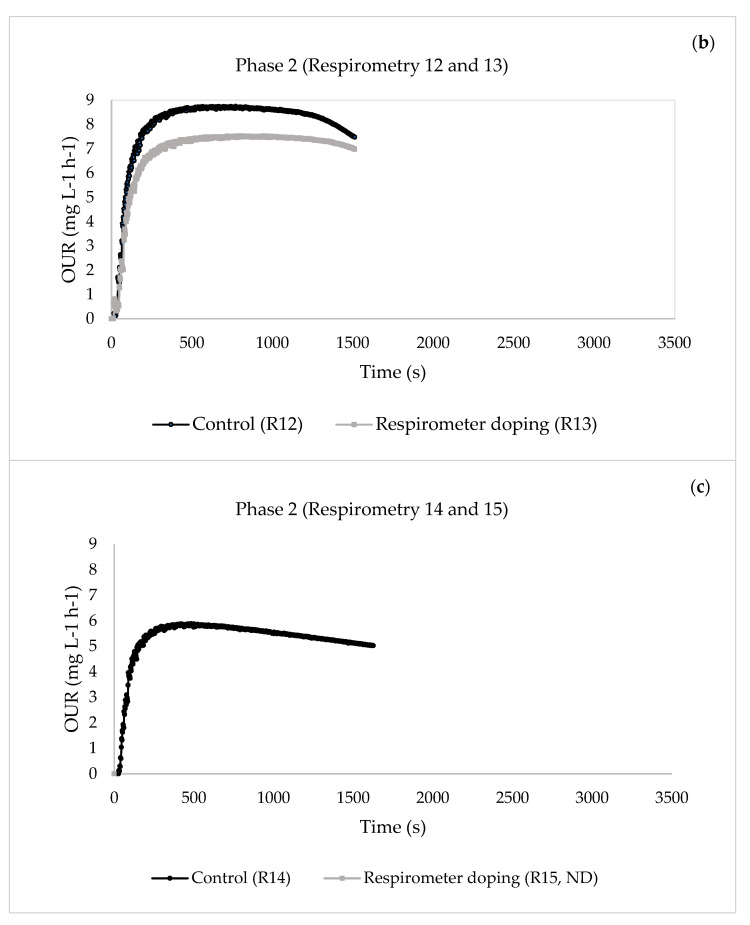
Evolution of the static oxygen uptake rate (OUR) in the respirometric experiments in Phase 2. (**a**) Phase 2: control (respirometric test 10) and pharmaceutical compound addition in the respirometer (respirometric test 11, not detected). (**b**) Phase 2: control (respirometric test 12) and pharmaceutical compound addition in the respirometer (respirometric test 13). (**c**) Phase 2: control (respirometric test 14) and pharmaceutical compound addition in the respirometer (respirometric test 15, not detected).

Regarding endogenous respiration, the results of the respirometric tests performed for the two phases are shown in Figure 4 and Figure 5.

In Phase 1, the presence of pharmaceutical compounds did not produce a notable variation of the maximum static oxygen uptake rate (OUR_end_) (Figure 4c,d), except for respirometric test 3 (Figure 4b), where a notable increase in this parameter was observed, with a change from 3.595 to 6.747 mg L^−1^ h^−1^. However, when the pharmaceutical concentration increased in Phase 2, a decrease in this parameter was observed (Figure 2b). This may have been caused by the effects of the pharmaceuticals on the heterotrophic biomass. Other authors studied the kinetic behaviour of MBR biomass against the compound erythromycin and found that the rates of oxygen consumption OUR and R_s_ decreased with increasing pharmaceutical concentration [39].

The variables R_s_ and OUR represented accelerated biochemical reactions. These trends were considered to evaluate the kinetic parameters, i.e., Y_H_, µ_max_, K_M_, and b_H_. Table 5 shows the kinetic parameters obtained for the respirometries during the two phases.

In Phase 1, with a lower pharmaceutical concentration, Y_H_ increased by 8.78% from control respirometry 1 to 0.6353 mg VSS mgDQO^−1^ in respirometry 2. When performing the pharmaceutical shock in respirometry 3, the amount of the heterotrophic biomass produced by oxidised substrate increased by 4.93%. The performance of Y_H_ with pharmaceutical intrusion was stable in the tests where no heterotrophic biomass was destroyed (0.64 ± 0.02 mg VSS mgCOD^−1^) (respirometries 3, 5, 7, and 13), which indicates that there was no statistically significant difference among the yield coefficients of the heterotrophic biomass. Other authors obtained Y_H_ values close to those obtained in this study for a kinetic study of MBR without the presence of pharmaceuticals [40].

In Phase 2 tests with higher concentrations of pharmaceuticals, the control respirometric tests 10 and 12 presented approximate values.

However, when the pharmaceuticals were added (respirometry 11), the system was inhibited. Nevertheless, upon the addition of pharmaceutical compounds (respirometry 11), the system encountered inhibition. However, in respirometry 13, the heterotrophic biomass demonstrated activity, likely due to prolonged exposure to the pharmaceutical compounds. However, starting from day 24 in phase 2, the system reached a point where it could no longer effectively withstand the impact of the pharmaceutical compounds, resulting in the cessation of its activity.

The adaptation of the plant was considered good because the determined values of Y_H_ did not present drastic decreases. Furthermore, when assessing the effects of the various concentrations of the studied pharmaceuticals, it was observed that lower concentrations exhibited comparatively lesser impacts, as evidenced by respirometries 3 and 7. In contrast, higher concentrations appeared to exert a more pronounced effect leading to a decline in the Y_H_ values, although this was not significant.

The substrate degradation rate of organic matter removal r_su,H_ decreased with pharmaceutical compound addition by 82.23% in Phase 2 (respirometric test 13) compared with the control value (respirometric test 12). There was also a decrease of 60.46% in Phase 1 respirometry 5 compared to respirometry 4. Therefore, degradation was slower in the presence of erythromycin, ibuprofen, and diclofenac in both phases.

The r_su,H_ value reported for respirometry 3, with the addition of pharmaceuticals, can be explained by the evolution of the dynamic oxygen consumption rate; the duration of the respirometric test was short (0.2 h). When comparing the two phases, it was observed that the reduction in r_su,H_ in Phase 1, characterized by lower pharmaceutical concentrations, was comparatively smaller following doping in the respirometer. This finding suggests a more favourable adaptation of the biomass to the conditions in Phase 1. Other authors studied the effects of ibuprofen and other compounds, one of an antibiotic nature, such as erythromycin, on the heterotrophic biomass of an MBR, also obtaining decreasing r_su,H_ values in the presence of these compounds [41].

Concerning the maximum specific growth rate μ_max_, for the shock at a high concentration of pharmaceuticals (Phase 2), there was a decrease in this parameter (respirometries 10, 12, 13, and 14). However, comparing respirometric tests 12 and 14, with 0.0732 and 0.0090 h^−1^, respectively, reductions in the maximum specific growth rate were also observed (pharmaceuticals at these higher concentrations can considerably affect the heterotrophic biomass). At more moderate concentrations of pharmaceuticals, a decrease in μ_max_ generally occurred from the third day of continuous doping onwards, reaching a value of 0.0101 h^−1^ at 17 days of doping (Phase 1, respirometry 6). Furthermore, in general, pharmaceutical shock also reduced the μ_max_ value. For respirometries 1 and 2, the μ_max_ decreased by 19.42%, from 0.0324 h^−1^ to 0.0272 h^−1^. When performing the pharmaceutical shock in respirometry 3, the maximum specific growth rate increased by 76.72%, reaching a value of 0.1167 h^−1^. In the subsequent respirometric tests 5 and 7, the μ_max_ value decreased again due to the addition of the pharmaceuticals.

The maximum specific growth rate μ_max_ followed a trend similar to that presented for r_su,H_, decreasing with the addition of pharmaceuticals, irrespective of the concentration and except for respirometry 3. In general, it progressively decreased in the presence of a mixture of pharmaceuticals in the urban wastewater. Whilst a more pronounced decrease was observed for higher pharmaceutical concentrations (respirometries 12, 13, and 14), the decrease in μ_max_ was less pronounced when the concentrations were lower.

Regarding the half-saturation coefficient of organic matter K_M_, the presence of pharmaceutical compounds caused a decrease in K_M_ and a progressive decrease in the constant in both tests for pharmaceutical concentrations corresponding to Phases 1 and 2, except for the respirometry control test 6, where there was an increase in 73.19% compared to the control. This decrease was again more pronounced in the trials with high pharmaceutical concentrations in the MBR.

These findings suggest that the presence of the pharmaceutical mixture implied a lower half-saturation of organic matter in urban wastewater treatment in the MBR, with this effect being more noticeable for high pharmaceutical concentrations. This indicates that less available substrate was required to reach μ_max_, suggesting that the MBR was not inhibited by the substrate but by the mix of the pharmaceuticals.

In the Phase 1 tests, a decrease in b_H_ was observed between respirometries 1 and 2, from 0.0725 to 0.0418 day^−1^, with a decrease in 73.60%. With respect to respirometry 3, when the pharmaceuticals were added to the respirometer, the b_H_ increased by 48.42%, reaching a value of 0.0825 day^−1^. This increase would justify the decrease in MLSS from 5633.33 to 3200 mg L^−1^. When the pharmaceutical shock occurred in respirometry 5, the b_H_ decreased by 4.82%. For respirometry 6, this trend was in line with a more pronounced reduction; immediately after the addition of the pharmaceuticals, it continued to decrease, reaching a value of 0.0346 day^−1^.

For Phase 2, in respirometries 10 and 12, the b_H_ value decreased from 0.1701 to 0.1389 day^−1^ by 22.40% and by an additional 18.79% in the presence of pharmaceutical compounds in respirometry 13, reaching a value of 0.1170 day^−1^, although in experiment 14, a slight increase in the coefficient was observed. This might explain the decrease in MLSS from day 17 (2433.33 mg L^−1^) to day 24 (1433.33 mg L^−1^). Other authors reported decreases in b_H_ values ranging from 24.76% to 66.51% in the kinetics of heterotrophic biomass in a membrane bioreactor against pharmaceutical compounds such as ciprofloxacin and carbamazepine [28]. Other authors conclude that heterotrophic biomass evolves and adapts to CECs at low concentrations [42], resulting in a kinetic response as in this study [43]. Consequently, it is of paramount importance to develop predictive models capable of mitigating elevated residual concentrations of pharmaceuticals prior to their discharge. This measure could effectively mitigate issues associated with the presence of pharmaceuticals compounds in WWTP and sludge-attaching compounds characterized by notably low biodegradability, such as diclofenac [16]. This proactive approach ensures proper treatment and prevents the release of these compounds into the environment.

## 4. Conclusions

In an MBR laboratory plant operating at an HRT of 24 h, fed with real urban wastewater, the heterotrophic biomass behaviour was analysed for two concentrations of the contaminants erythromycin, ibuprofen, and diclofenac. The concentrations studied for the first phase were erythromycin 0.576 mg L^−1^, ibuprofen 0.056 mg L^−1^, and diclofenac 0.948 mg L^−1^. For phase 2, the concentrations were increased to erythromycin 1.440 mg L^−1^, ibuprofen 0.140 mg L^−1^, and diclofenac 2.370 mg L^−1^. The conclusions were as follows:

- Heterotrophic biomass was affected and inhibited by the presence of pharmaceutical drugs in both phases of the operation. The system response to low concentrations of pharmaceutical compounds occurred in the initial phase. However, after 22 days of doping, the system response was inhibited. At the highest concentrations of pharmaceuticals, the system was not able to react, and the response was not activated; activity was completely inhibited after 11 days of doping.
- Under the operating conditions of this study, there was a gradual decrease in the concentration of MLSS (from 5633 to 2133 mg L^−1^ for Phase 1 and from 2400 to 500 mg L^−1^ for Phase 2) and in the removal of COD (from 87% to 28% in Phase 1 and from 65% to 20% in Phase 2) in the system as it was not able to absorb the effect produced by the pharmaceutical compounds added in both phases.
- For Phase 1, when the concentrations of the pharmaceuticals were lower, the substrate degradation rate of organic matter removal r_su,H_ increased from 11.4626 to 44.7977 mg O_2_ L^−1^ h^−1^ after four days of continuous doping, indicating that the system could actively mitigate the impact of the pharmaceuticals. However, the value decreased from day 5 of doping until the system was inhibited. This inactivation of the heterotrophic biomass was seen in the reduction of the percentage of COD elimination, which decreased from an initial 92% to 28%. The same behaviour was observed for Phase 2, where inhibition of the system occurred earlier than in Phase 1 because the pharmaceutical compounds had a greater effect on the heterotrophic biomass due to their higher concentrations.
- For future investigations, it is advisable to incorporate the assessment of the removal performance of the dosed pharmaceuticals in the laboratory plant, along with the monitoring of phosphorus in its different forms and nitrogen removal, as well as the corresponding related compounds. Such comprehensive analyses would contribute to a more comprehensive understanding of the overall treatment efficiency and environmental impact of the pharmaceutical compounds in the system.

## Figures and Tables

**Table 1:** Concentrations of pharmaceuticals in both operation phases.

Pharmaceutical	Phase 1 (mg L^−1^)	Phase 2 (mg L^−1^)
Erythromycin	0.576	1.440
Diclofenac	0.948	2.370
Ibuprofen	0.056	0.140

**Table 2:** Permeability working values. TMP: transmembrane pressure.

			Pressure (bar)	∆TMP	Permeability (m^3^/(m^2^ h bar))	Permeability (L/(m^2^ h bar))
Phase 1	Day 0–15	Suction	0.08	0.93	0.002039	2.04
		Backwashing	0.20	0.80	0.002358	2.36
	Day 16–35	Suction	0.28	0.73	0.002602	2.60
		Backwashing	0.20	0.80	0.002358	2.36
Phase 2	Day 0–15	Suction	0.10	0.90	0.001395	1.39
		Backwashing	0.30	0.70	0.001793	1.79
	Day 16–35	Suction	0.05	0.95	0.001321	1.32
		Backwashing	0.25	0.75	0.001674	1.67

**Table 3:** Respirometric tests corresponding to Phases 1 and 2.

Phase	Operation Time (Day)	Respirometric Test
1	13	1—Control
	17	2—Control
		3—Respirometer doping
	23	4—Control
		5—Respirometer doping
	30	6—Control
		7—Respirometer doping
	35	8—Control
		9—Respirometer doping
2	13	10—Control
		11—Respirometer doping
	17	12—Control
		13—Respirometer doping
	24	14—Control
		15—Respirometer doping
	35	16—Control
		17—Respirometer doping

**Table 4:** Water characterisation and organic matter removal. MLSS: mixed liquor suspended solids; COD: chemical oxygen demand.

Phase	Day	MLSS (mg/L)	COD Influent (mgO_2_/L)	COD Removal (%)	BOD_5_ Removal (%)	pH Influent	pH Effluent	pH Bioreactor	Conductivity Influent (µS/cm)	Conductivity Effluent (µS/cm)	Conductivity Bioreactor (µS/cm)
1	0–12	5394 ± 421	483 ± 22	92 ± 2	91 ± 1	7.86 ± 0.08	7.72 ± 0.32	7.63 ± 0.41	1306 ± 50	1223 ± 78	1295 ± 73
	13	5633	549	87	82	8.02	8.01	7.78	1485	1138	1172
	17	3200	306	81	79	8.01	8.24	7.91	1503	1370	1445
	23	3900	100	80	65	7.54	8.53	6.96	1517	1601	1292
	30	3933	329	66	56	7.74	8.38	6.49	1378	1364	1200
	35	2133	323	28	21	8.00	6.63	6.97	1310	1287	1291
2	0–12	5145 ± 1286	434 ± 8	72 ± 3	65 ± 4	7.70 ± 0.20	7.82 ± 0.69	7.7 ± 0.75	791 ± 52	662 ± 57	705 ± 47
	13	2400	524	65	57	7.84	7.26	7.00	1071	732	826
	17	2433	534	52	44	8.06	7.90	8.15	1410	1242	1268
	20	1233	349	36	29	8.31	6.78	6.93	1239	1002	1016
	22	1967	309	21	15	8.21	5.88	6.31	1225	966	977
	24	1433	496	22	14	8.11	6.00	6.74	1174	936	968
	35	500	420	20	14	7.38	7.25	8.26	1408	1085	1220

**Table 5:** Kinetic parameters of the heterotrophic biomass. Y_H_: yield coefficient of heterotrophic biomass; µ_max_: maximum specific growth rate of heterotrophic biomass; K_M_: half-saturation coefficient of organic matter (K_M_); b_H:_ decay coefficient of heterotrophic biomass; r_su,H_: substrate degradation rate of organic matter removal.

Phase	Operation Time (Day)	Respirometric Test	Y_H_ (mgVSS/mgO_2_)	μ_max_ (h^−1^)	K_M_ (mgO_2_/L)	b_H_ (day^−1^)	r_su,H_ (mgO_2_/Lh)
1	13	1—Control	0.5907	0.0324	20.5560	0.0725	11.4626
	17	2—Control	0.6353	0.0272	11.6292	0.0418	10.8555
		3—Respirometer doping	0.6683	0.1167	11.4102	0.0825	44.7977
	23	4—Control	0.6585	0.0090	1.5089	0.0475	12.7022
		5—Respirometer doping	0.6415	0.0052	1.3799	0.0453	7.9161
	30	6—Control	0.6541	0.0101	5.6285	0.0357	8.3688
		7—Respirometer doping	0.6624	0.0054	1.1072	0.0346	12.2834
	35	8—Control	ND	ND	ND	ND	ND
		9—Respirometer doping	ND	ND	ND	ND	ND
2	13	10—Control	0.6620	0.2492	38.9945	0.1701	15.3234
		11—Respirometer doping	ND	ND	ND	ND	ND
	17	12—Control	0.6413	0.0732	13.9664	0.1389	17.4938
		13—Respirometer doping	0.6270	0.0152	4.6379	0.1170	9.5998
	24	14—Control	0.6423	0.0090	1.0064	0.1411	5.5038
		15—Respirometer doping	ND	ND	ND	ND	ND
	35	16—Control	ND	ND	ND	ND	ND
		17—Respirometer doping	ND	ND	ND	ND	ND

ND: not detected.

## Data Availability

Not applicable.
